# Supplementary material for: Molecular basis of convergent evolution of ACE2 receptor utilization among HKU5 coronaviruses
Source: bioRxiv. 2024 Aug 28:2024.08.28.608351. Preprint. [Version 1] doi: 10.1101/2024.08.28.608351 (PMC11383307; doi:10.1101/2024.08.28.608351)
Supplement: Supplement 1 — Figure S1. HKU5 utilizes several mammalian ACE2s as receptors. (A-D) Binding of the HKU5-19s RBD-hFc to (A-B) and entry of HKU5-19s S VSV pseudovirus into (C-D) HEK293T cells transiently transfected with the indicated bat (A, C) or non-bat (B, D) mammalian ACE2 orthologs. Scale bars: 100 μm. Data are shown as the MEAN ± SD for C-D. n=3 biological replicates. Data representative of two independent experiments for A-B, and a single experiment for C-D. Figure S2. CryoEM data processing of the P.abr ACE2-bound HKU5 RBD dataset (A-B) Representative electron micrograph and 2D class averages of the complex embedded in vitreous ice. Scale bars: 100 nm (A) and 150 Å (B). (C) Gold-standard Fourier shell correlation curve. The 0.143 cutoff is indicated by a horizontal dashed line. (D) Local resolution estimation calculated using cryoSPARC and plotted on the sharpened map. (E) Data processing flowchart. CTF: contrast transfer function; NUR: non-uniform refinement. The angular distribution of particle images calculated using cryoSPARC is shown as a heat map. Figure. S3. Structural comparisons of the receptor binding domain of HKU5 with HKU4, NeoCoV and MERS-CoV. (A-D) Comparison of the structures of the P.abr ACE2-bound HKU5 RBD (PDB 9D32, this study), the P.pip ACE2-bound NeoCoV RBD (PDB 7WPO39), hDDP4-bound MERS-CoV RBD (PDB 4KR048) and hDDP4-bound HKU4 RBD (PDB 4QZV38). (E) Superimposition of the HKU5 (cyan), NeoCoV (orange-red), MERS-CoV (pink) and HKU4 (green) RBMs. Figure S4. Biolayer interferometry analysis of the P.abr ACE2 ectodomain binding to the immobilized HKU5-19s and HKU5-33s RBDs. Binding avidities were determined by steady state kinetics and are reported as apparent affinities (KD, app). One representative out of two technical replicates is shown Fig S5. Dose-response curves of HKU5-19s VSV pseudovirus neutralization by MERS-CoV infection-elicited plasma or monoclonal antibodies. A, Dose-response curves of HKU5-19s VSV pseudovirus neutralization by MERS-CoV infe [file NIHPP2024.08.28.608351v1-supplement-1.pdf]

bioRxiv preprint doi: https://doi.org/10.1101/2024.08.28.608351; this version posted August 28, 2024. The copyright holder for this preprint (which was not certified by peer review) is the author/funder, who has granted bioRxiv a license to display the preprint in perpetuity. It is made available under aCC-BY 4.0 International license.

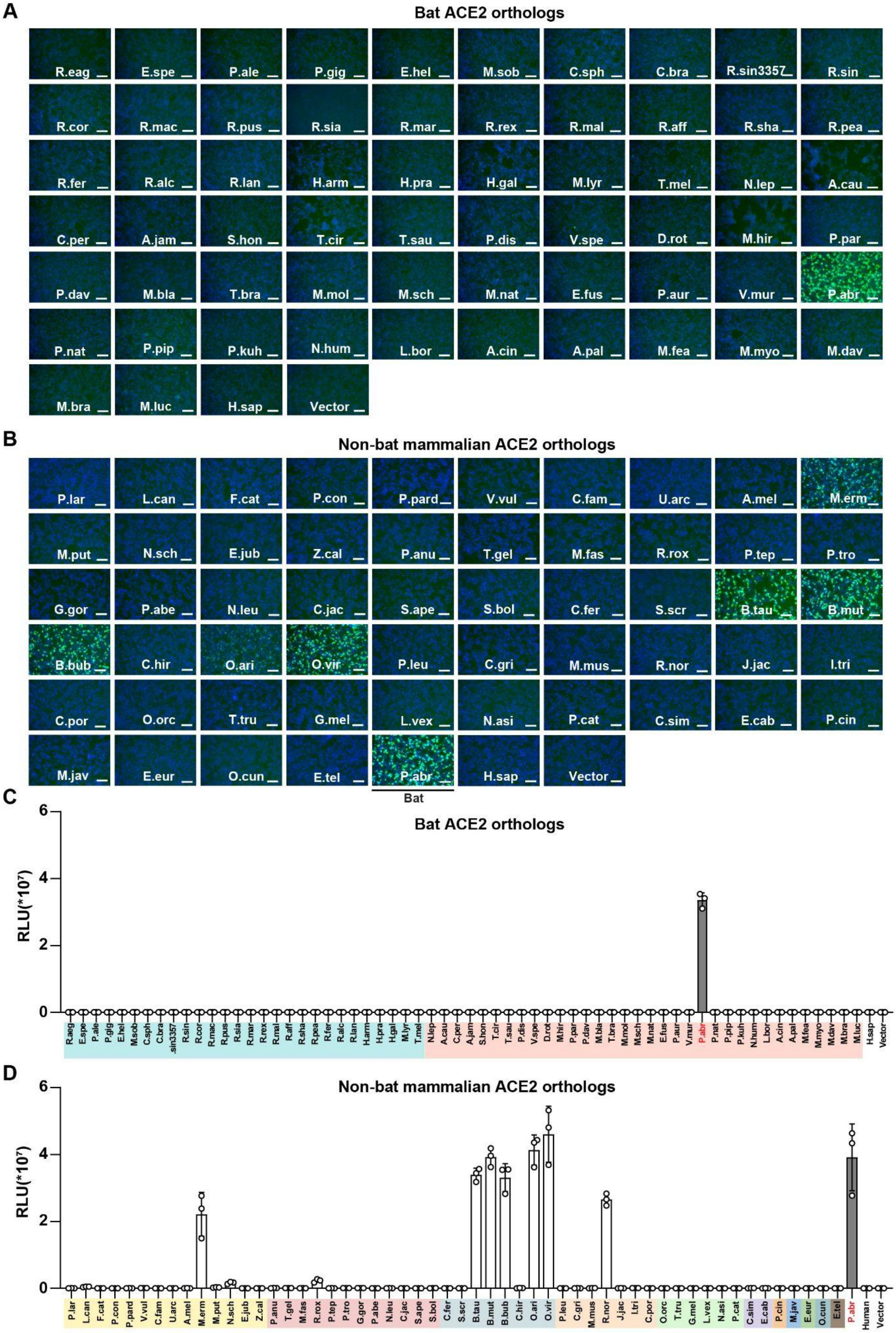

bioRxiv preprint doi: https://doi.org/10.1101/2024.08.28.608351; this version posted August 28, 2024. The copyright holder for this preprint (which was not certified by peer review) is the author/funder, who has granted bioRxiv a license to display the preprint in perpetuity. It is made available under aCC-BY 4.0 International license.

Figure S1. HKU5 utilizes several mammalian ACE2s as receptors. (A-D) Binding of the HKU5-19s RBD-hFc to (A-B) and entry of HKU5-19s S VSV pseudovirus into (C-D) HEK293T cells transiently transfected with the indicated bat (A, C) or non-bat (B, D) mammalian ACE2 orthologs. Scale bars: 100 μm. Data are shown as the MEAN ± SD for C-D. n=3 biological replicates. Data representative of two independent experiments for A-B, and a single experiment for C-D.

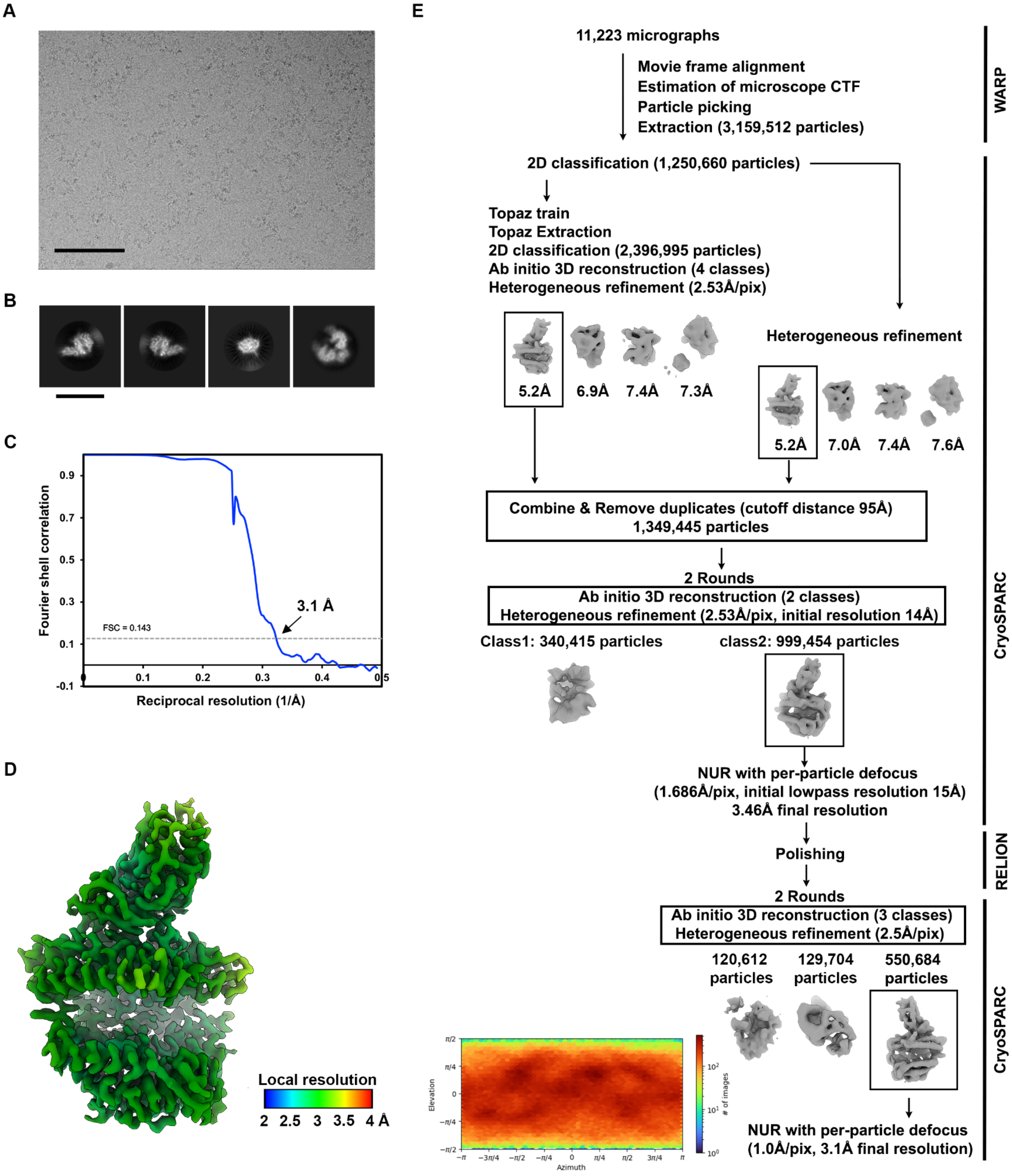

bioRxiv preprint doi: https://doi.org/10.1101/2024.08.28.608351; this version posted August 28, 2024. The copyright holder for this preprint (which was not certified by peer review) is the author/funder, who has granted bioRxiv a license to display the preprint in perpetuity. It is made available under aCC-BY 4.0 International license.

Figure S2. CryoEM data processing of the P.abr ACE2-bound HKU5 RBD dataset (A-B) Representative electron micrograph and 2D class averages of the complex embedded in vitreous ice. Scale bars: 100 nm (A) and 150 Å (B). **(C)** Gold-standard Fourier shell correlation curve. The 0.143 cutoff is indicated by a horizontal dashed line. **(D)** Local resolution estimation calculated using cryoSPARC and plotted on the sharpened map. **(E)** Data processing flowchart. CTF: contrast transfer function; NUR: non-uniform refinement. The angular distribution of particle images calculated using cryoSPARC is shown as a heat map.

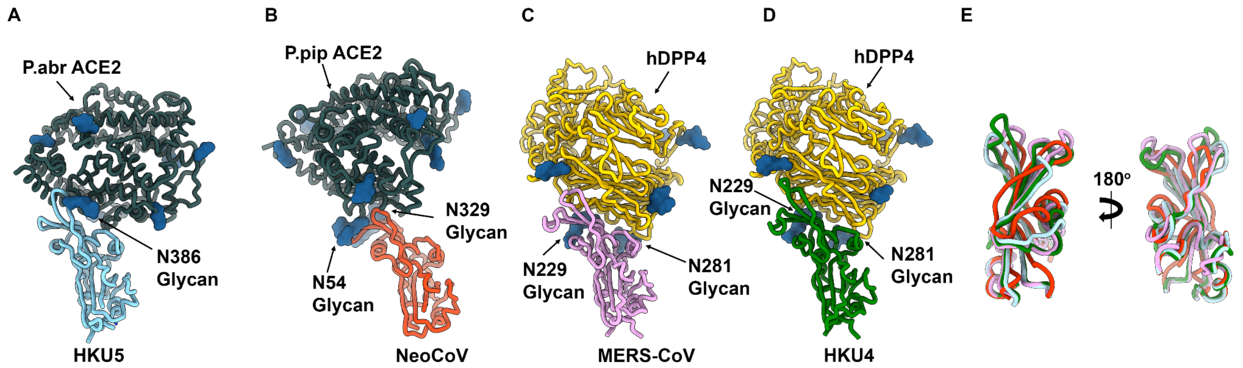

Figure. S3. Structural comparisons of the receptor binding domain of HKU5 with HKU4, NeoCoV and MERS-CoV. (A-D) Comparison of the structures of the P.abr ACE2-bound HKU5 RBD (PDB 9D32, this study), the P.pip ACE2-bound NeoCoV RBD (PDB 7WPO[39]), hDDP4-bound MERS-CoV RBD (PDB 4KR0[48]) and hDDP4-bound HKU4 RBD (PDB 4QZV[38]). **(E)** Superimposition of the HKU5 (cyan), NeoCoV (orange-red), MERS-CoV (pink) and HKU4 (green) RBMs.

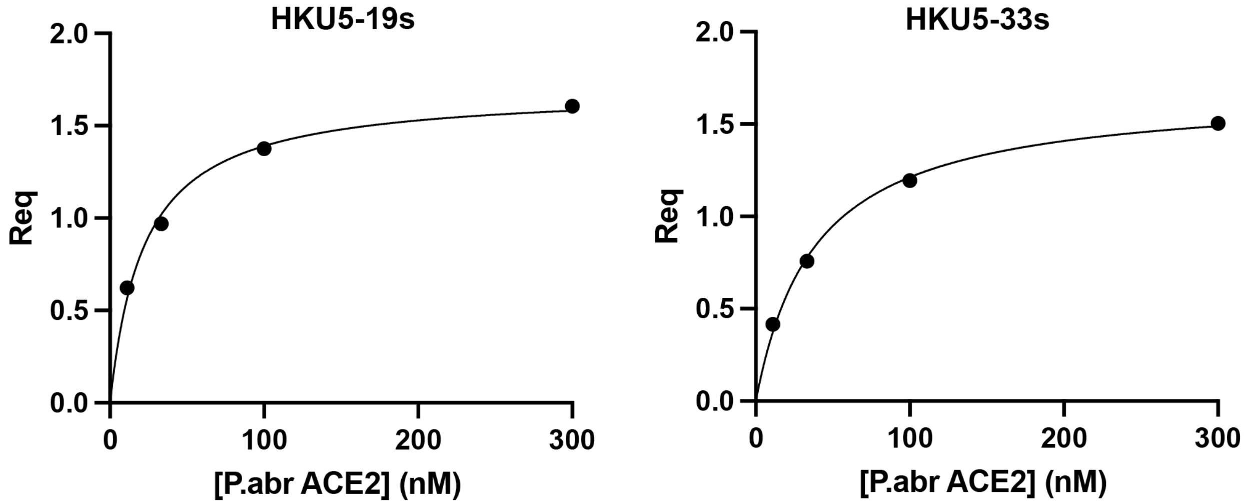

Figure S4. Biolayer interferometry analysis of the P.abr ACE2 ectodomain binding to the immobilized HKU5-19s and HKU5-33s RBDs. Binding avidities were determined by steady state kinetics and are reported as apparent affinities (K_D, app). One representative out of two technical replicates is shown

bioRxiv preprint doi: https://doi.org/10.1101/2024.08.28.608351; this version posted August 28, 2024. The copyright holder for this preprint (which was not certified by peer review) is the author/funder, who has granted bioRxiv a license to display the preprint in perpetuity. It is made available under aCC-BY 4.0 International license.

A

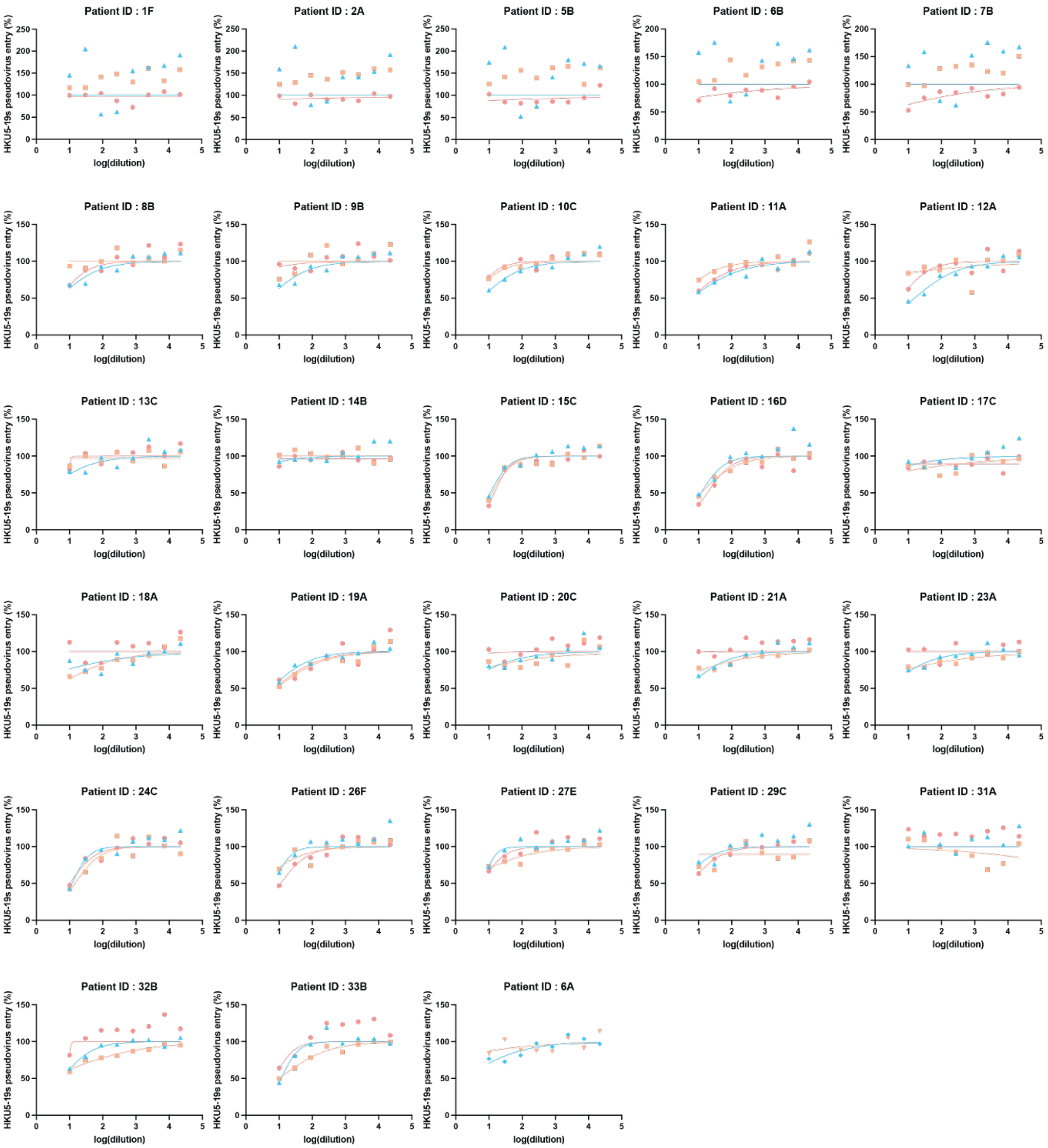

B

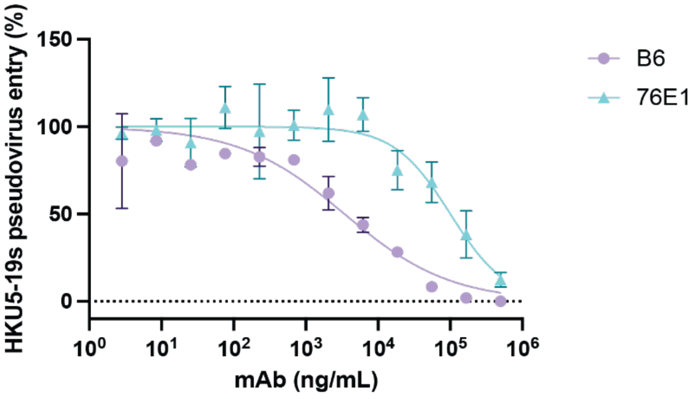

bioRxiv preprint doi: https://doi.org/10.1101/2024.08.28.608351; this version posted August 28, 2024. The copyright holder for this preprint (which was not certified by peer review) is the author/funder, who has granted bioRxiv a license to display the preprint in perpetuity. It is made available under aCC-BY 4.0 International license.

Fig S5. Dose-response curves of HKU5-19s VSV pseudovirus neutralization by MERS-CoV infection-elicited plasma or monoclonal antibodies. A, Dose-response curves of HKU5-19s VSV pseudovirus neutralization by MERS-CoV infection-elicited plasma. Each data point represents the mean of two technical replicates and three biological replicates with three distinct batches of pseudoviruses are shown with different colors. **B**, Dose-response curves of HKU5-19s VSV pseudovirus neutralization by fusion machinery-directed monoclonal antibodies. Each data point represents the mean of two technical replicates. A representative curve from three biological replicates is shown.

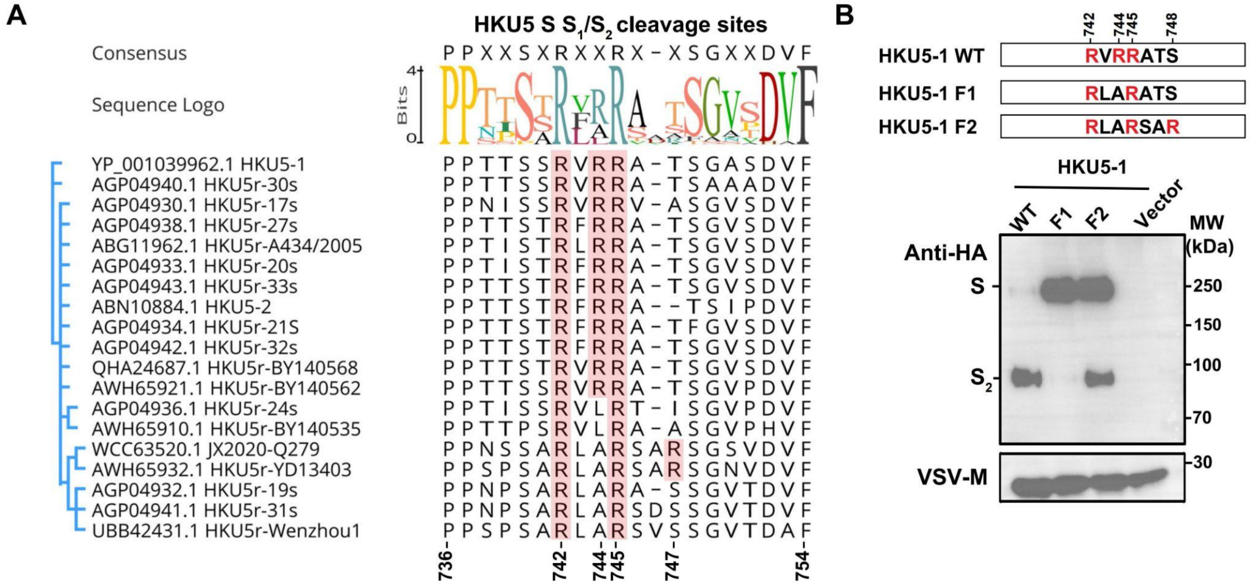

Figure S6. HKU5 S proteolytic processing during biogenesis.

A, Sequence analysis of S glycoprotein S_1/S_2 junction from the indicated HKU5 isolates, with arginine (R) residues highlighted with red background. **B**, Quantification of S glycoprotein proteolytic processing and incorporation in VSV pseudoviruses of wildtype and S_1/S_2 HKU5-1 mutants analyzed by Western blot detecting the C-terminal-fused HA tag**.** VSV-M was used as a loading control. HKU5-1 S residue numbering is shown.
